# Evaluation of the effectiveness of multi‐task cognitive activation therapy combining motor and cognitive tasks in patients with schizophrenia

**DOI:** 10.1002/pcn5.70137

**Published:** 2025-06-10

**Authors:** Junichi Kino, Tsubasa Morimoto, Yasuhiro Matsuda, Masato Honda, Toshifumi Kishimoto, Takashi Okada

**Affiliations:** ^1^ Department of Psychiatry Nara Medical University School of Medicine Kashihara Japan; ^2^ Department of Psychiatry Akitsukounoike Hospital Gose Japan; ^3^ Department of Psychiatry Asuka Hospital Takatori Japan; ^4^ Department of Psychiatry Osaka General Medical Center Osaka Japan; ^5^ Department of Psychiatry Osaka Phychiatric Medical Center Osaka Japan; ^6^ Department of Psychiatry Shichiyama Hospital Kumatori Japan

**Keywords:** cognition, group therapy, multi‐task, physical exercise, schizophrenia

## Abstract

**Aim:**

To examine the effects of a multi‐task cognitive activation therapy (MCAT) program combining motor and cognitive tasks in patients with schizophrenia.

**Methods:**

Patients with schizophrenia who received psychiatric day care treatment were included in this study. The study used a mirror‐image test with a 3‐month pre‐intervention and intervention period each. MCAT training was conducted twice a week for 12 weeks for a total of 24 sessions. The Brief Assessment of Cognition in Schizophrenia ‐ Japanese version (BACS‐J) and Facial Emotional Identified Test were used for the primary outcome; the secondary outcomes included the Positive and Negative Syndrome Scale (PANSS), and the Life Assessment Scale for the Mentally Ill (LASMI).

**Results:**

Thirty‐six patients completed the intervention. The *Z*‐score of the BACS‐J before and after the intervention period included verbal memory (−1.98 ± 1.60 before intervention, −1.50 ± 1.41 after intervention, *P* < 0.01, *r* = 0.47) and motor function (−1.47 ± 1.71 before intervention, −0.93 ± 1.50 after intervention, *P* < 0.01, *r* = 0.47). Significant improvement was observed in the composite score (−2.31 ± 1.51 before intervention, −1.92 ± 1.38 after intervention, *P* < 0.01, *r* = 0.52). Significant improvements were also observed on the PANSS comprehensive psychopathology scale and the LASMI interpersonal relationships scale. No other endpoints demonstrated significant improvements. The BACS‐J composite score, which was the earliest BACS‐J examination, was considered the baseline for all the participants in the moderate and severe groups. The severe group (*n* = 22) demonstrated significant improvements in the BACS‐J verbal memory, composite score, and LASMI interpersonal relationships.

**Conclusion:**

These results suggest that the MCAT may improve cognitive function and interpersonal relationships in patients with schizophrenia and severe or moderate cognitive impairment.

## INTRODUCTION

Most patients with schizophrenia experience cognitive dysfunction,^1^, ^2^ resulting in challenges in their daily lives, such as taking time to learn new things and being unable to perform personal tasks such as cleaning and cooking. Furthermore, cognitive dysfunction makes it challenging to concentrate on a task, remember the steps for completing a task, and pay attention to the surrounding environment, thus affecting academic and work performance and functional outcomes of people with schizophrenia.^3^, ^4^ Therefore, rehabilitation programs for cognitive dysfunction have attracted much attention, with many reported studies on their ameliorative effects.^5^, ^6^, ^7^ Cognitive rehabilitation programs include a variety of computerized and non‐computerized programs, and their effectiveness warrants the consideration of individual patient characteristics.^8^ Some patients with schizophrenia have no experience with computers and are unable to adapt to computerized programs, therefore we decided to implement a program that uses physical exercise as an easily accessible approach for daily clinical use.

Previous studies have reported the effects of exercise programs on improving cognitive function.^9^ This is attributed to an increase in the systemic blood flow and the promotion of brain‐derived neurotrophic factor (BDNF) by exercise.^10^, ^11^ Exercise also contributes to improvements in basic physical fitness and health promotion. In Japan, exercise programs are readily available in psychiatry, as sports are often included in occupational therapy and psychiatric day‐care treatment programs.

Recently, multitask programs, such as Life Kinetic^12^ for athletes, and CogniSize^13^ and Synapsology^14^ for the elderly in Japan are also attracting attention. A multitask program comprises tasks that simultaneously involve physical exercise and cognitive training. Interventions combining aerobic exercise and cognitive tasks have been reported in patients with schizophrenia,^15^ but interventions using multiple tasks have not yet been reported. Therefore, we aimed to create a multitask program that is easily accessible to patients with schizophrenia in their daily clinical practice and investigate whether it could contribute to improvement in the cognitive function and psychiatric symptoms.

We previously developed multitask cognitive activation therapy (MCAT), a multitask program for motor and cognitive tasks in patients with schizophrenia.^16^ Training was designed to be administered in pairs for ease of use in clinical settings. The basic movements comprised throwing and passing a ball and beanbag to and from a paired partner. These low‐impact physical exercises were supplemented with multiple cognitive tasks. Moreover, a task for reading the paired partner's feelings was included in the “reflection” section after the training, which was intended to activate the participants' social cognition.

We conducted two previous studies: a 6‐month pre‐ and post‐intervention comparative study^17^ and a 12‐month mirror‐image study.^18^ In the latter mirror ‐ image study, 13 patients with schizophrenia were administered the MCAT, which resulted in improvements in attention, processing speed, and interpersonal interaction skills in cognitive function. In the other study, the MCAT was administered to seven patients with schizophrenia; although no improvement was observed in the cognitive function, attention promotion was observed to a paired partner. However, the intervention period in both previous studies was 6 months, which was long, resulting in the dropout of many participants. A 3‐month cognitive rehabilitation intervention for patients with schizophrenia has been reported to improve cognitive function,^19^, ^20^ therefore in this study we decided to examine the effectiveness of a shortened version of the MCAT with an intervention period of 3 months in patients with schizophrenia.

## METHODS

### Participants

The participants included individuals diagnosed with schizophrenia according to the International Statistical Classification of Diseases and Related Health Problems (ICD‐10). Their ages ranged from 20 to 65 years and they included patients receiving psychiatric day‐care treatment. Those with orthopedic conditions and with a Global Assessment of Functioning score of ≤39 were excluded from the study. The sample size was calculated from the effect size using G*Power (Department of Psychology at the University of Düsseldorf in Germany, version 3.1.9.7). Based on an effect size (*d*) of 0.32 obtained from the sample size in Takano's previous study,^17^ an effect size (*r*) of 0.57 was obtained. From this effect size (*r*), with a power of 0.95 and a significance level (*α*) of 0.05, the sample size required for this study was 30 participants. The study sites included psychiatric hospitals in Japan, Nara Medical University, and four private psychiatric hospitals. Posters specifying the eligibility criteria for this study were displayed and participants were recruited.

### MCAT contents

MCAT is a program developed to activate cognitive function by performing multiple tasks that combine motor and cognitive tasks. Participants are paired with one another, and multiple pairs (actually 2–4 pairs) can participate simultaneously. The program is conducted twice a week for 40 min each time. The schedule comprised 5 min for orientation, 25 min for performing multiple tasks, and 10 min for reflection. The rules for participation included (1) enjoying head confusion and failure, (2) loudly expressing movements, voices, and emotions, and (3) observing the partner carefully (Table 1).

**Table 1:** Rules for participating in MCAT.

1	Enjoy the confusion of your mind.
2	It's okay if you're not good at it, so just have fun with a smile.
3	Don't compete with others, and enjoy failure.
4	Speak clearly and loudly.
5	Approach the task with big actions.
6	Express your feelings as much as possible.
7	Match the MCAT partner's pace.
8	Find something good about the MCAT partner.

Abbreviation: MCAT, multi‐task cognitive activation therapy.

### Intervention procedure

During the orientation, all the participants reviewed the purpose of the MCAT and the rules of participation. The participants subsequently decided on their training partners (MCAT partners). The aim of choosing an MCAT partner is to activate participants' social cognition by having them carefully observe their partner. One of the four disciplines was conducted per session. There were four events: three events played in pairs and one event played in groups (Table 2). In group Circle Pass, three or four pairs are in a circle. If the number of participants is small, staff join the group. In Circle Pass, participants choose a partner and carefully observe their MCAT partner while completing the task.

**Table 2:** Overview and examples of MCAT tasks.

Task name	Overview and examples
Beanbag Catch 1	Participants are divided into commanders and players and stand facing each other. The commander throws the beanbag. The players catch the beanbag while following the commander's instructions and saying the words. Example: When the commander says “right,” catch the beanbag with your right hand, and at the same time step forward with your left foot and say the name of a vegetable.
Beanbag Catch 2	Stand facing your MCAT partner. Work in sync with your MCAT partner to toss the beanbags you are each holding at the same time, then catch them while counting out loud the number of tosses. Example: When the number of tosses is a multiple of three, throw the beanbag straight up in the air and say the name of the animal at the same time.
Basket Soccer Rugby	Participants are divided into commanders and players. They stand facing each other. The commander gives one of three commands: “Basket,” “Soccer,” or “Rugby.” Players throw the ball back using the movement that is instructed. Example: When the commander gives the command “Basket,” players throw the ball back using the shooting form for a basketball while simultaneously saying a word beginning with “A.”
Circle Pass	Five to six participants form a circle facing each other. Two colored balls are used, and the action of throwing the ball and the way of speaking change depending on the color. Example: When the red ball comes around, throw it while saying the last name of the next member who will pass it. When the blue ball comes around, roll it while saying the first name of the next member who will pass it.

Abbreviation: MCAT, multi‐task cognitive activation therapy.

**Figure 1:**
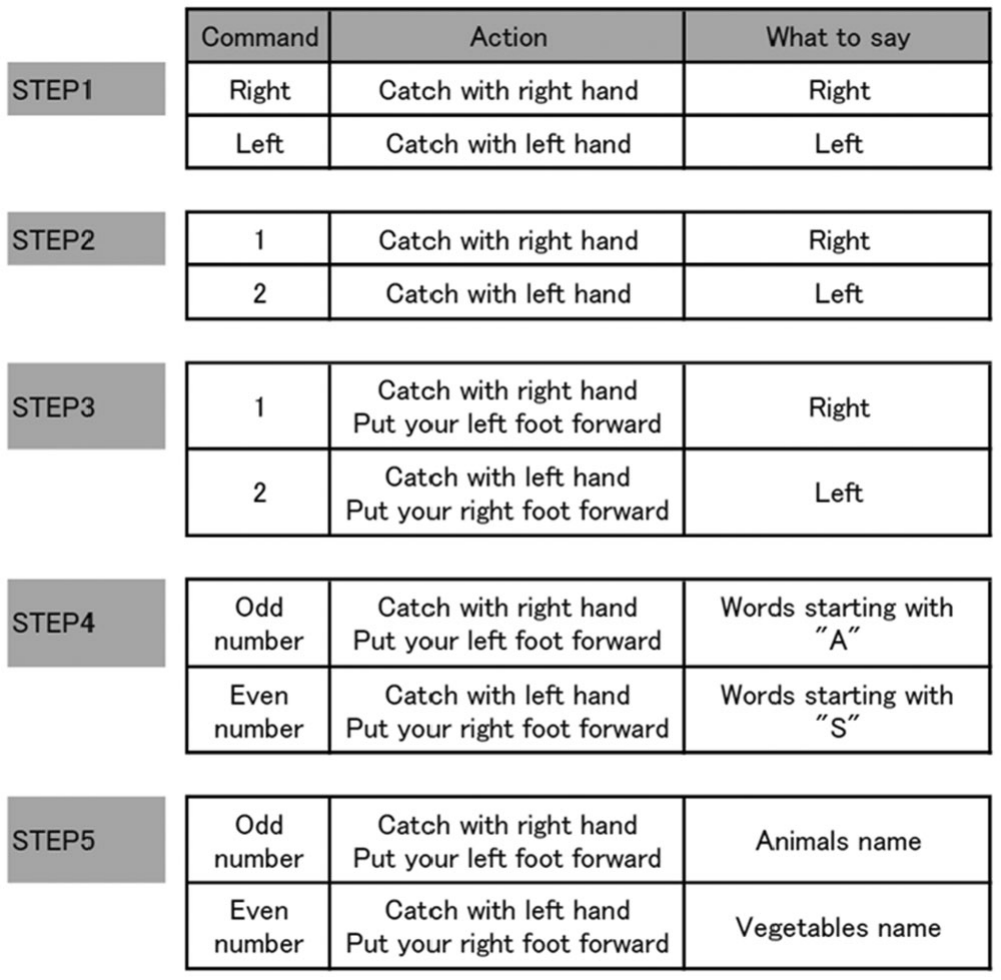
Example of difficulty settings for multiple tasks Beanbag Catch 1”. “Command” refers to the content of the command issued by the commander. “Action” refers to how the player moves their body in response to the command issued. “What to say” refers to the verbal expression used by the player in response to the commands issued.

In each event, multiple tasks were performed by adding these three tasks according to the manual: (1) additional limb movements, (2) modified instructions, and (3) additional cognitive tasks to the basic movements, such as throwing and catching a ball or a cue ball. In the first step, simple movements, such as throwing and catching a ball or beanbag, were performed. After the second step, motor tasks, such as complex limb movements and stepping movements, and cognitive tasks, such as verbal fluency tasks, were added step‐by‐step to increase the difficulty level of the multiple tasks. In Beanbag Catch 1 and Basket Soccer Rugby, one of the MCAT partners is the player and the other is the commander. Each step lasts for 2 min. After completing steps 1 to 6, the players and commanders switch roles. In Beanbag Catch 2 and Circle Pass, there is no division into players and commanders, and everyone is a player. An implementation manual was created to ensure that the degree of difficulty did not vary according to the therapist who facilitated the tasks. A specific example of the difficulty setting for multiple tasks is shown in Figure 1. The setting was designed to activate neurocognition comprehensively by memorizing multiple tasks, paying attention to the instructions, and instantaneously executing appropriate physical movements. Because these multiple motor and cognitive tasks are performed simultaneously and instantaneously, it is difficult for anyone to perform them perfectly. The participants are repeatedly told that “everyone makes mistakes” and “it is important that members laugh at each other's mistakes and enjoy working on them.”

**Figure 2:**
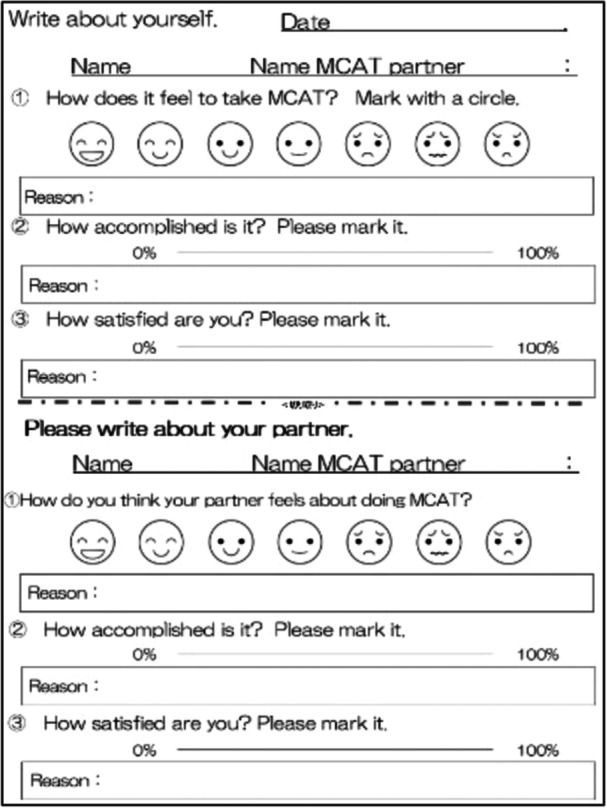
MCAT sharing sheet. The MCAT sharing sheet is an A4 sheet of paper with a two‐tiered structure. In the top half, participants write down the mood, achievement, and satisfaction they felt after performing the MCAT. In the bottom half, participants predict and write down the mood, achievement, and satisfaction their MCAT partner will feel after performing the MCAT. MCAT, multi‐task cognitive activation therapy.

During MCAT sharing, time was set aside for the trained partners to review their feelings towards each other. For this MCAT sharing, an original MCAT sharing sheet was used (Figure 2). The sheet has columns for selecting the degree of each partner's mood, achievement, and satisfaction while performing the task and the reason for the selection. The upper and lower halves of the sheet contained the same questions, with the upper half describing oneself and the lower half predictably describing one's partner. The sheet determined the specific area of the partner to be observed. After completing the descriptions, the participants were seated across their partners and presented their descriptions to each other.

### Study design

A mirror‐image test was used in this study. There was a 3‐month period without intervention (pre‐intervention period) and a 3‐month period in which the multi‐task MCAT intervention was implemented (intervention period). In this study, the intervention period was set at 3 months owing to the many dropouts in a previous study with an intervention period of 6 months. Three time points were set for measuring the effectiveness: initial evaluation (Time 1), intermediate evaluation (immediately before the intervention, Time 2), and final evaluation (immediately after the intervention, Time 3).

### Measurements

The primary outcomes included the Brief Assessment of Cognition in Schizophrenia‐Japanese version (BACS‐J)^21^, ^22^ for assessing neurocognitive function, and the Facial Emotional Identified Test (FEIT)^23^ for assessing social cognitive function. Secondary outcomes included the Positive and Negative Syndrome Scale (PANSS) to assess psychiatric symptoms, the Life Assessment Scale for the Mentally Ill (LASMI) to assess social functioning, the Recovery Assessment Scale (RAS)^24^ to assess recovery, the Basic Psychological Need Satisfaction and Frustration Scale (BPNSFS)^25^ to assess intrinsic motivation, and the Keio Version Wisconsin Card Sorting Test (WCST) to assess neurocognitive function.

### BACS‐J

BACS‐J is an assessment tool evaluating six neurocognitive functions. Scores were calculated using *Z*‐scores and compared with the average scores of healthy participants. A score that is exactly the same as the average score of healthy participants is zero and cognitive decline is indicated by a negative *Z*‐score.

### FEIT

The FEIT is an evaluation of a participant's cognitive function of facial expressions, and determines whether the facial expressions of a person displayed on a personal computer screen express joy, sadness, fear, anger, surprise, disgust, or no emotion. The score was 21 points, and the percentage of correct answers was determined for each of the six emotions.

### RAS

The RAS is a questionnaire‐based assessment that grades recovery questions on a 5‐point scale with five sub‐items; the higher the score, the better.

### PANSS

The PANSS is a rating scale that assesses the severity of psychiatric symptoms based on the observations and interviews of patients with schizophrenia.

### LASMI

The LASMI is an observational rating index that comprehensively captures the status of life disturbances in individuals with mental disorders. It has five subitems, with lower scores indicating less disturbance.

### BPNSFS

The BPNSFS assesses the degree to which the basic psychological needs of autonomy, competence, and relationships are satisfied or frustrated; 24 questions, four items for satisfaction and four items for frustration with each need were included in the test. Each item was graded on a 7‐point Likert scale.

### WCST

The WCST is a neuropsychological test that measures cognitive function, particularly flexibility, problem solving, and executive function. This test measures the examinee's ability to adapt to rule changes. In this study, we used the KWCST (keio version), which can be administered on a personal computer.^26^, ^27^ In this study, the KWCST calculated (1) the number of consecutive correct responses achieved six times (categories achieved: CA), (2) the number of response cards used until the first category is achieved (numbers of response cards until the first category [NUCA]), (3) total errors (TE), (4) perseverative errors of Milner (PEM), the number of erroneous responses that were still classified into the same category as the last achieved category despite the change of category, (5) the number of responses classified into the same category as the last erroneous response (PEM), (6) the number of erroneous responses classified into the same category as the previous erroneous response (perseverative error of Nelson [PEN]), and (7) the number of erroneous responses that occurred after two or more but less than five consecutive correct responses (difficulties in maintaining set [DMS]).

### Statistical analyses

Multiple comparisons were performed for the results at the initial evaluation (Time 1), intermediate evaluation (Time 2), and final evaluation (Time 3). Each dataset was tested for normality using the Shapiro–Wilk test, with corresponding *t*‐tests performed for the tests for which normality was confirmed for Time 1 and Time 2, and for Time 2 and Time 3. Wilcoxon signed‐rank sum tests were performed for test items for which normality could not be confirmed. To perform the paired *t*‐test or Wilcoxon signed‐rank sum test twice, Bonferroni correction was applied and the significance level was adjusted to 2.5%. The statistical software used was R4.4.1 (R Foundation for Statistical Computing).

### Ethical compliance

This study was approved by the Research Ethics Committee of Nara Medical University and was conducted after the participants were informed verbally and in writing about the purpose and methods of the study, following which written informed consent was obtained.

## RESULTS

### Participants' characteristics

Basic information about the participants is presented in Table 3. Assuming dropouts, consent was obtained from 44 participants. Of these, 36 (20 males and 16 females, mean age 42.7 ± 11.8 years) were able to complete the intervention. Of the eight dropouts, five withdrew their consent before the MCAT intervention and three were hospitalized due to physical illness during the MCAT intervention.

**Table 3:** Participant characteristics (*n* = 36, male:female = 20:16).

Characteristic	Mean	Standard deviation	Range
Age (years)	42.7	11.8	22–63
Years of education	12.6	2.2	9–16
Age at onset (years)	23.4	8.5	12–46
Age at first visit (years)	24.1	9.6	12–59
Untreated period (years)	0.8	2.2	0–12
Duration of illness (years)	18.4	12.1	2–45
Number of hospitalizations	2.8	2.8	1–13
Length of hospital stay (months)	43.5	65.8	1–276

The results of the BACS‐J are presented in Table 4. Among the *Z*‐scores of the BACS‐J during the intervention period, verbal memory (Time 2, −1.98 ± 1.60; Time 3, −1.50 ± 1.41; P < 0.01, *r* = 0.47), motor function (Time 2, −1.47 ± 1.71; Time 3, −0.93 ± 1.50; P < 0.01, *r* = 0.47) and composite score (Time 2, −2.31 ± 1.51; Time 3, −1.92 ± 1.38; P < 0.01, *r* = 0.52) demonstrated statistical significance.

**Table 4:** Changes in the Brief Assessment of Cognition in Schizophrenia (BACS‐J) scores of participants (*n* = 36) during the pre‐intervention and intervention periods.

Parameter	Time 1	Time 2	Time 3	*t*‐test			
				Time 1 vs. Time 2		Time 2 vs. Time 3	
*n* = 36	Mean (SD)	Mean (SD)	Mean (SD)	*P*	*r*	*P*	*r*
Verbal memory	−2.19 (1.67)	−1.98 (1.60)	−1.50 (1.41)	0.18	0.23	<0.01*	0.47
Working memory	−1.72 (1.47)	−1.80 (1.45)	−1.66 (1.33)	0.51	0.11	0.20	0.22
Motor function	−1.00 (1.33)	−1.47 (1.71)	−0.93 (1.50)	0.07	0.30	<0.01*	0.47
Verbal fluency	−1.50 (1.10)	−1.37 (0.98)	−1.38 (1.04)	0.20	0.22	0.91	0.02
Attention processing speed	−1.54 (1.14)	−1.41 (1.14)	−1.27 (1.25)	0.21	0.21	0.16	0.24
Executive function	−0.60 (1.46)	−0.56 (1.39)	−0.36 (1.17)	0.89	0.02	0.26	0.19
Composite sore	−2.29 (1.66)	−2.31 (1.51)	−1.92 (1.38)	0.89	0.02	<0.01*	0.52

Abbreviations: SD, standard deviation; Time 1, 3 months before intervention; Time 2, immediately the before intervention; Time 3, immediately after the intervention.

In the WCST and FEIT, the results of the 24 participants who were able to correctly perform the three tests were analyzed; in the WCST, in the preintervention period, CA (Time 1, 3.13 ± 2.05; Time, 2 4.08 ± 1.67; P < 0.01, *r* = 0.83), TE (Time 1, 20.46 ± 8.83; Time 2, 17.29 ± 8.15; P = 0.02, *r* = 0.35) demonstrated statistical significance. No significant improvement was noted in the FEIT (Time 1, 14.65 ± 3.10; Time 2, 14.50 ± 2.64; Time 3, 14.56 ± 2.88).

Other assessment measures demonstrated significant improvement in the overall psychopathology scale of the PANSS (Time 2, 30.86 ± 7.07; Time 3, 28.17 ± 7.00; P < 0.01, *r* = 0.30) and in the interpersonal relationship part of the LASMI (Time 2, 0.90 ± 0.70; Time 3, 0.63 ± 0.49; P < 0.01, *r* = 0.18). No significant improvement was observed in RAS (Time 2, 82.17 ± 15.27; Time 3, 83.33 ± 14.74; P = 0.44, *r* = 0.21). The subscales of the BPNSFS were autonomy satisfaction (Time 2, 13.25 ± 3.70; Time 3, 13.19 ± 3.23; P = 0.90, *r* = 0.02), autonomy frustration (Time 2, 10.83 ± 3.59; Time 3, 9.42 ± 3.55; P = 0.04, *r* = 0.05), relatedness satisfaction (Time 2, 14.56 ± 3.15; Time 3, 14.31 ± 3.35; P = 0.52, *r* = 0.11), relatedness frustration (Time 2, 10.58 ± 3.20; Time 3, 9.36 ± 3.35; P = 0.06, *r* = 0.08), competence satisfaction (Time 2, 11.94 ± 3.82; Time 3, 11.73 ± 3.81; P = 0.02), and competence frustration (Time 2, 11.50 ± 3.59; Time 3, 10.42 ± 3.17; P = 0.06, *r* = 0.31).

**Figure 3:**
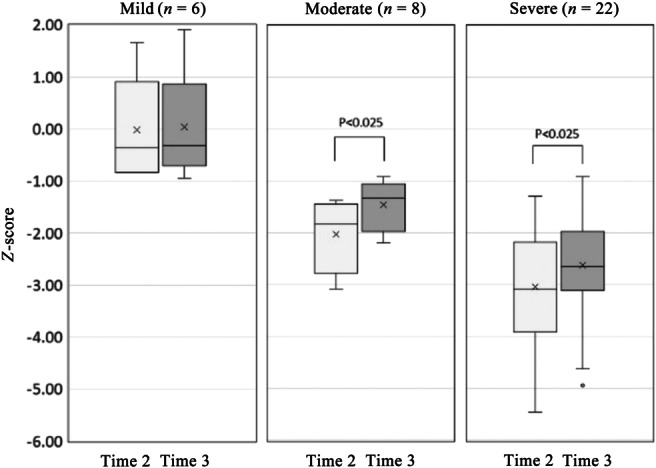
Changes in the Brief Assessment of Cognition in Schizophrenia Composite Score at Time 2 and Time 3 according to severity of cognitive impairment.

All the participants were grouped by cognitive severity using the BACS‐J Composite Score at Time 1 as the baseline. The *Z*‐score criteria for each group were −1.0 or higher for the mild group, −1.0 to −2.0 for the moderate group, and −2.0 or lower for the severe group.^28^ There were 6, 8, and 22 patients in the mild, moderate, and severe groups, respectively (Figure 3). The results of Time 2 and Time 3 in the BACS‐J composite score for each group are shown in Figure 3. The *Z*‐scores for the mild group (age 31.2 ± 13.5, five males and one female) were −0.01 and 0.04 for Time 2 and Time 3, respectively. When the moderate and severe groups, for which statistical analysis was possible, were analyzed, the moderate group (age 45.5 ± 13.3, four males and four females) demonstrated significant improvement in the BACS‐J composite score (Time 2, −2.02 ± 0.68; Time 3, −1.45 ± 0.47; P < 0.025, *r* = 0.89). The severe group (age 44.8 ± 9.2, 11 males and 11 females) demonstrated significant improvement in the BACS‐J verbal memory (Time 2, −2.84 ± 1.01; Time 3, −2.15 ± 1.05, P < 0.01, *r* = 0.59), composite score (Time 2, −3.04 ± 1.13; Time 3 −2.62 ± 1.07, P < 0.025, *r* = 0.49), LASMI Interpersonal Relationships (Time 2, 13.00 ± 9.40; Time 3, 9.41 ± 6.96; P < 0.025, *r* = 0.07), and PANSS Composite Psychopathology Scale scores (Time 2, 32.7 ± 7.09; Time 3, 28.7 ± 7.43; P < 0.025, *r* = 0.25). There was no significant improvement in the motor function measured by BACS‐J (Time 2, −1.71 ± 1.57; Time 3, −1.09 ± 1.27; P = 0.05, *r* = 0.52).

## DISCUSSION

This study aimed to test the efficacy of a 3‐month MCAT as a new cognitive rehabilitation program for patients with schizophrenia. The MCAT intervention in this study resulted in significant improvements in the BACS‐J verbal memory, motor function, composite score, PANSS total psychopathology scale score, and LASMI interpersonal relationships. The moderate group demonstrated significant improvement in the BACS‐J composite score. The severe group demonstrated significant improvement in the verbal memory of the BACS‐J, composite score, interpersonal relations of LASMI, and overall PANSS psychopathology scale score.

Factors contributing to verbal memory impairment in patients with schizophrenia are multifaceted and include neurobiological factors,^29^ symptoms of schizophrenia,^30^ and abnormalities in the brain regions associated with memory.^31^ Interventions for improving verbal memory include auditory training to directly promote verbal memory.^32^ MCAT requires memorization of multiple tasks with increasing difficulty. The participants (players) listened carefully to the instructions provided by the commander, memorized the instructions, and then recalled and performed the multiple memorized tasks. This memory‐to‐execution sequence is considered trained verbal memory. In cognitive rehabilitation interventions, verbal memory has been identified as a partial mediator of functional improvement and plays an important role in enhancing performance‐based functioning in schizophrenia.^33^ Other studies have reported that verbal memory demonstrated improvements by interventions adding exercise to cognitive training.^34^ Improvement in verbal memory and not working memory suggests that our training included content that required longer retention and more complex instructional comprehension than working memory.

Factors contributing to motor dysfunction in patients with schizophrenia are multifaceted and include neurodevelopmental and neural connectivity deficits.^35^, ^36^ Abnormalities in the cerebellum, which play an important role in motor control and regulation, have been implicated in motor dysfunction in patients with schizophrenia.^37^, ^38^ In the dual task, the manual dexterity of patients with schizophrenia is presumably more impaired than that in a single task, affecting their performance in daily life.^39^ The motor task used in the MCAT involves simple movements, such as throwing, kicking, rolling, and catching, using a ball and a cue ball. This task requires the use of motor control functions, such as speed, timing, height, and angle, to make it easier for the opponent to catch the ball. In addition to the upper limb movements to handle the ball and plectrum, multiple tasks require the simultaneous execution of lower limb movements. It is possible that the repetition of multiple tasks involving these physical movements activated motor control and motor regulation functions in the cerebellum and improved BACS‐J motor function. The activation of verbal memory and motor function may have also contributed to significant improvements in the BACS‐J composite score. The BACS‐J results were similar for all participants and the severe group owing to the large number of participants in the severe group. The mild group did not show any MCAT effect due to their higher baseline *Z*‐scores. Further investigations are warranted to determine the effects of cognitive impairment on disease severity. However, no significant improvement was observed in the WCST scores following the intervention. The significant improvement before the start of the intervention (Time 2) may have been attributed to the learning effect, which has been reported previously,^40^, ^41^ and the sense of evaluation every 3 months may have been too short.

Interpersonal interaction skills in patients with schizophrenia are affected by impaired social cognition,^42^ negative symptoms,^43^ and neurocognitive impairment.^44^ In the present study, we examined the effects of MCAT on social cognition. The FEIT was used as the assessment measure, but no significant improvement was observed. Patients with schizophrenia show significant impairments in the recognition of facial emotions than healthy controls.^45^, ^46^ We expected that our program would activate facial expression recognition function in participants because they would be paired face‐to‐face with a partner and could observe the partner's facial expressions; however, we could not confirm this effect. Direct training focused on facial expression recognition is important for improving facial expression recognition in patients with schizophrenic^47^; however, our program did not aim to promote facial expression recognition, although the participants were instructed to focus on the facial expressions of their paired partner. This may be the reason for this difference. On the other hand, communication with the paired partner and the presence for time for “reflection” after the multiple tasks facilitated interpersonal interaction and significantly improved LASMI interpersonal relationships. This is considered a significant improvement in the LASMI interpersonal relationships.

Cognitive rehabilitation in patients with schizophrenia has been reported to improve negative and depressive symptoms.^48^, ^49^ Patients with schizophrenia have overly negative beliefs and attitudes about their ability to engage in goal‐directed behavior, referred to as defeatist performance beliefs (DPBs), which affect social outcomes by interfering with motivation and goal‐directed behavior.^50^ To mitigate DPBs, MCAT has established “enjoy failure” as one of its rules. This experience may have contributed to the improvement in the PANSS Comprehensive Psychopathology Scale scores by restoring self‐esteem and self‐efficacy. Exercise is also a useful adjunct therapy for improving psychiatric symptoms in patients with schizophrenic and is said to reduce psychiatric symptoms.^51^ MCAT uses low‐impact physical exercise, and mild‐to‐moderate‐impact exercise has been reported to improve psychiatric symptoms,^52^ therefore this may have influenced the improvement in the PANSS scores.

We expected that the RAS and BPNSFS scores would also improve as participants enjoyed the challenges and multiple tasks. However, the current study found no significant improvement in the RAS and BPNSFS scores; RAS scores are presumably affected by personal wishes, control, and relationships with others.^53^ According to the BPNSFS, the higher the rate of social dysfunction in patients with schizophrenia, the stronger the level of frustration.^54^ Thus, RAS and BPNSFS scores are presumably influenced by the participant's social life and environmental factors, therefore an effect with the MCAT, which focuses only on cognitive function and interpersonal interaction, may not have been observed; the MCAT activates cognitive function and interpersonal interaction. It is performed using familiar tools, such as balls and beanbags, and does not require special qualifications. Because it can be implemented with low‐impact exercises, the program is easy to participate in and is easy to apply clinically.

## LIMITATIONS AND FUTURE DIRECTIONS

This study has several limitations. First, this was a multicenter study, and uniformity in the setting and quality of data collection across sites may not have been fully achieved. Second, the wide age range of the participants made it difficult to fully control for age‐related effects. Third, this study was not a randomized controlled trial, which limits its ability to rigorously corroborate causality, therefore these results should be interpreted with caution. The results of this study suggest that MCAT may be effective for schizophrenic patients with severe cognitive impairment, therefore focused research on the profile of patients with schizophrenia for whom MCAT is effective is warranted.

## CONCLUSION

Rehabilitation with MCAT for patients with schizophrenia activates verbal memory, motor function, and overall cognitive function. The MCAT is a psychosocial program that is amenable to clinical applications.

## AUTHOR CONTRIBUTIONS

**Junichi Kino:** Data acquisition and analysis; drafting the manuscript. **Tsubasa Morimoto:** Data acquisition and analysis; revising the manuscript. **Yasuhiro Matsuda:** Data acquisition and analysis; revising the manuscript. **Masato Honda:** Data acquisition and analysis; revising the manuscript. **Toshifumi Kishimoto:** Supervision. **Takashi Okada:** Supervision. All authors read and approved the final draft.

## CONFLICT OF INTEREST STATEMENT

The authors declare no conflict of interest.

## ETHICS APPROVAL STATEMENT

This study was approved by the Ethics Review Committee of Nara Medical University (Approval No. 2132).

## PATIENT CONSENT STATEMENT

Written informed consent was obtained from all the study participants.

## CLINICAL TRIAL REGISTRATION

UMIN 000039704.

## Data Availability

The data that support the findings of this study are available from the corresponding author, Junichi Kino, upon reasonable request.
